# Impact of ethical leadership on work environment in public hospital in Saudi Arabia: A cross-sectional study

**DOI:** 10.12669/pjms.42.2.13804

**Published:** 2026-02

**Authors:** Badr K. Aldhmadi, Rakesh Kumar, Bilesha Perera, Mohammad A. Algarni

**Affiliations:** 1 Dr. Badr K. Aldhmadi, Ph.D. Department of Health Management, College of Public Health and Health Informatics, University of Ha’il, Ha’il, Saudi Arabia; 2 Dr. Rakesh Kumar, Ph.D. Department of Health Management, College of Public Health and Health Informatics, University of Ha’il, Ha’il, Saudi Arabia; 3 Dr. Bilesha Perera, MSc., Ph.D. Faculty of Medicine, University of Ruhuna, Galle, Sri Lanka. Department of Health Management, College of Public Health and Health Informatics, University of Ha’il, Ha’il, Saudi Arabia; 4 Dr. Mohammad A. Algarni, Ph.D. Faculty of Economic and Administration, King Abdulaziz University, Jeddah, Saudi Arabia

**Keywords:** Ethical Leadership, Work environment, Public Hospital, Saudi Arabia, PLS-SEM

## Abstract

**Background and Objective:**

At present, ethical outrages and moral disputes are major concerns in healthcare organizations. In such situations, ethical behavior has emerged an important enabler to manage healthcare organizations. There are several beneficial outcomes that have been documented in the growing body of research on ethical leadership. Ethical behavior would cause a positive effect not only on followers’ attitudes and behaviors but also on work environment in healthcare organizations. The study intended to assess the impact of ethical leadership on work environment in public hospital in Hail, Saudi Arabia.

**Methodology:**

In this crosse sectional study, a total of 387 working health care professionals in public hospitals in Hail, Saudi Arabia filled the distributed questionnaire between March 9, 2025 to May 14, 2025. Study respondents were selected through a simple random sampling method. Version- 4 of Smart PLS was used to apply partial least squares-structural equation modeling (PLS-SEM) to validate conceptual model.

**Results:**

In this study 55.3% of the respondents were women, 44.7% were men, and the average years of experience was 8.7 years. Study results found the statistically significant direct effect of ethical leadership on work environment (β=0.850, t=39.168, P<0.001).

**Conclusion:**

The study revealed that ethical leadership have positive impact on work environment in public hospitals in Hail, Saudi Arabia.

## INTRODUCTION

Currently, patients and society have higher expectations from healthcare organizations and professionals to fulfil social responsibility, support public health issues, acknowledge patients’ rights and basic human decency, and guarantee care with compassion and respect. Worldwide, healthcare organizations have implemented a multiple-tier management structure to meet stakeholders’ expectations. Executive officers who manage public hospitals are usually responsible for formulating the policies and the middle layer of the organization implements these policies. Ethical outrages and moral disputes are major concerns of healthcare organizations at present.^1^ Thus, organizations in the healthcare sector require employees with appropriate management and leadership skills.^2^ It was observed that hospitals are taking certain decisions about leadership styles and other leadership-related issues in order to improve healthcare service delivery. Hospitals need an integrated leadership approach that considers the workforce’s economic, social, psychological, and cultural differences while making personal decisions.^3^

Ethical behavior has become an important issue in healthcare management.^4^ Employees are more likely to trust and follow their managers if the workplace is fair and ethical.^5^ A leader can motivate and make measures to retain their team members, but it is pretty challenging if an employee who is unethical, dishonest, or unreliable is retained.^6^ Concurrently, it was seen that an ethical manager encourage good behavior by using a system of rewards and punishments to show what is good and bad.^7^ Ethical leadership creates an organization’s culture of honesty, integrity, and loyalty.^8^ Moreover, ethical leadership has a substantial positive influence on healthcare personnel’s work engagement.^9^ Langlois et al. (2014) suggested that an ethical leader develops a conducive working environment for the employees.^10^

Bai, Lin, & Liu conducted studies on ethical leadership and suggested to check the findings of their study in other nations also.^11^ Similarly, Hassan, Mahsud, Yukl, & Prussia urged that findings on the association of ethical leadership with other outcome dimensions be expanded or replicated, as well as to evaluate whether ethical leadership exhibits consistent results in different settings and nations.^12^ Even, the literature shows that there are only few studies^13^-^15^ conducted on ethical or value-based leadership in Saudi Arabia, particularly in the healthcare sector. Hence, this study focused on examining the impact of ethical leadership on the work environment in public hospitals. Thus, we presume:

*H_1_:* Ethical leadership has an impact on the work environment in public hospital contexts.

## METHODOLOGY

**Fig.1:**
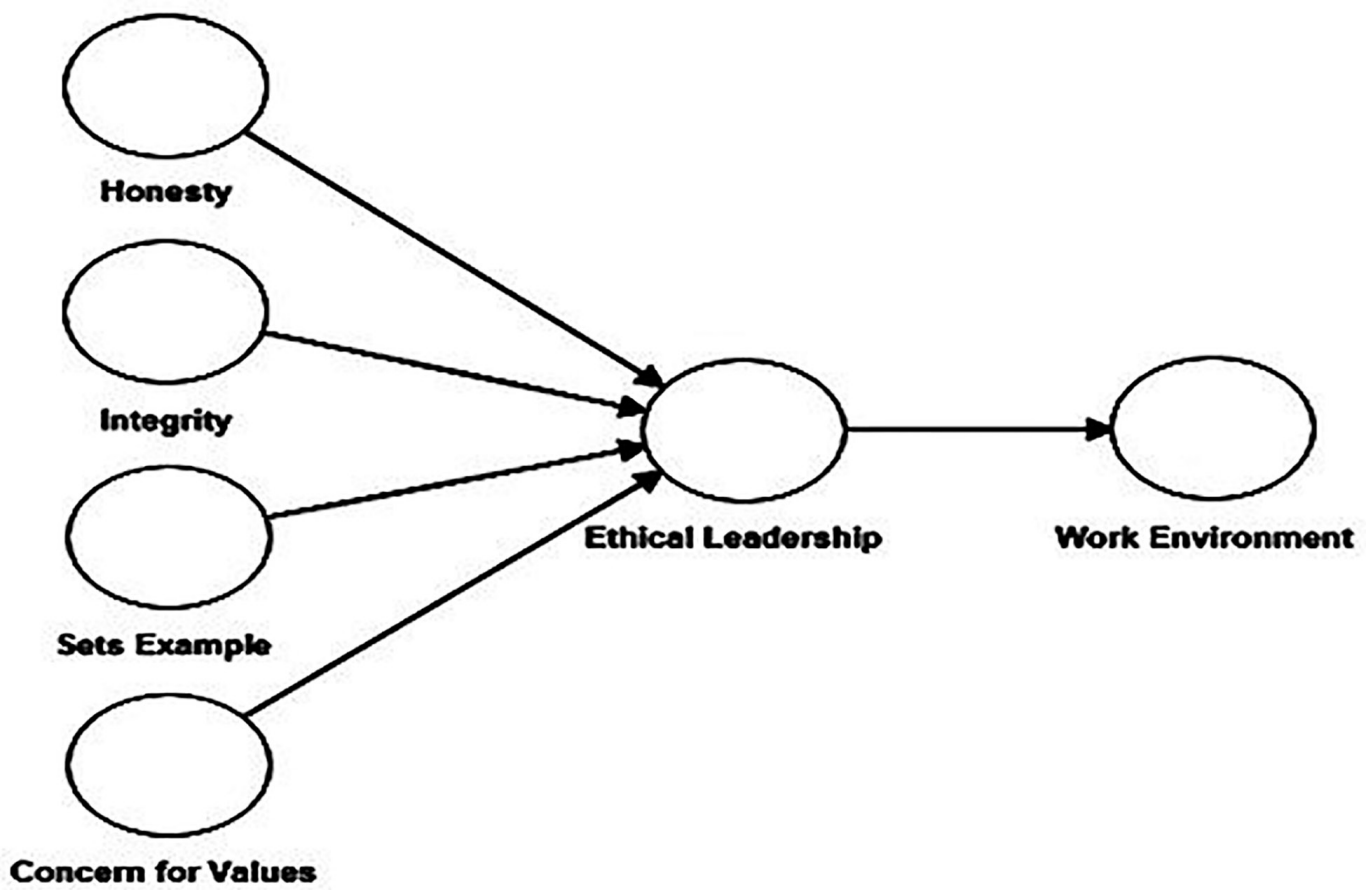
Conceptual Model.

In this study, a cross-sectional research design approach was adopted. The researcher tried to examine the impact of ethical leadership on the work environment in public hospitals. These two constructs were assessed using six anchor measurement scales that have been previously examined and verified. Badr et al in his study used Ethical Leadership Questionnaire and proposed four ethical leadership factors i.e. honesty, integrity, sets example and concern for values.^16^ The current study used these four factors to measure ethical leadership construct (Fig.1). Hence, conceptual model comprises reflective formative second-order construct and reflective first-order construct (Fig.1) was developed and validated.

A 20-item questionnaire was employed to collect data from the respondents. The questionnaire includes 15 items (honesty=4items, integrity=4items, sets example=3items and concern for values=4items) adopted from Ethical Leadership Questionnaires^17^ scale to study reflective formative second-order constructs and five items were included to study the reflective first-order construct. An adopted questions were initially built in English. To ensure that respondents fully understood the questions, the Arabic translation of the English version of the questionnaire was also conducted. To know the comprehensibility and understandability of the questionnaire, it was assessed by 8 working healthcare professionals in public hospitals in Hail city.

The study respondents were the working health care professionals in public hospitals in Hail, Saudi Arabia. This study employed a simple random sampling method to select study respondents. A total four hundred seventy (470) questionnaires were distributed among the respondents from March 9, 2025 to May 14, 2025. Out of the 470 distributed questionnaires, 387 were filled. Hence, the response rate was 82.34%. The respondents received an informed consent form during data collection, which described the study’s goal and included an explanation of the objective.

The present study used two stage approach to assess the measurement models. In the first stage, reliability, convergent validity, and discriminant validity of reflective dimensions of higher order construct (i.e. Ethical leadership) and the reflective lower order construct (i.e. work environment) were assessed. To validate reliability, the items’ outer loadings, Cronbach’s Alpha, and composite reliability (CR) have to be more than 0.70 and convergent validity is confirmed by the average variance extracted (AVE), which also to be greater than 0.50.^18^,^19^ HTMT ratio value lower than 0.85 or 0.90 used to confirm discriminant validity.^20^ In the second stage, the measurement models of one reflective-formative higher order construct were assessed.^21^ To assess multicollinearity in the indicators of higher order construct, all the VIF values should be less than 3. Additionally, the outer weight should be significant.^19^,^22^ Further, partial least squares-structural equation modeling (PLS-SEM) was employed to test the hypotheses of this study by using SMART PLS (version 4) software. The exploratory characteristics of the PLS-SEM and its intricate conceptual framework have made it the appropriate method for our paper.^21^,^22^ Sample adequacy was checked prior performing PLS-SEM analysis. PLS-SEM may be performed with a sample size of 100.^23^ As a result, collected data is sufficient to do the analysis.

### Ethical Approval:

Before commencing this study, ethical approval were obtained from University of Ha’il Research Ethics Committee (Ethics Review Number: H-2020-196) and Ministry of Health (IRB Registration Number: H-08-L-074) in Saudi Arabia.

## RESULTS

### Model Assessment:

This study used two stages approach to assess the measurement model. In the first stage, reliability and validity were assessed for five reflective exogenous constructs (i.e. honesty, integrity, sets example, concern for values and work environment). To assess the reliability and convergent validity of these five reflective measurement models, items’ outer loadings for each construct, Cronbach’s Alpha, composite reliability (CR), and average variance extracted (AVE) were measured. All the constructs possess outer loadings, Cronbach’s Alpha, and CR values higher than 0.70 and Average Variance Extracted (AVE) values higher than 0.50 (Table-I). Hence, construct reliability and convergent validity were confirmed. Further, heterotrait-monotrait (HTMT) ratio approach employed to measure the discriminant validity. The results (Table-II) indicate the establishment of discriminant validity.

**Table-I:** Measurement Model: Items’ outer loading, reliability and convergent validity.

	Λ	α	CR	AVE
Honesty (H)		0.873	0.914	0.726
H1	0.894			
H2	0.871			
H3	0.858			
H4	0.780			
Integrity (I)		0.850	0.899	0.691
I1	0.783			
I2	0.803			
I3	0.859			
I4	0.875			
Sets Example (SE)		0.892	0.933	0.822
SE1	0.910			
SE2	0.913			
SE3	0.897			
Concern for Value (CV)		0.853	0.901	0.694
CV1	0.845			
CV2	0.862			
CV3	0.822			
CV4	0.802			
Work Environment (WE)		0.872	0.907	0.662
WE1	0.779			
WE2	0.827			
WE3	0.770			
WE4	0.843			
WE5	0.845			

***Note 1:*** See Appendix 1 for full items.

***Note 2:*** Λ = Outer loadings, α = Cronbach’s Alpha, CR = Composite Reliability, AVE = Average Variance Extracted.

**Table-II:** Discriminant validity by using HTMT.

Constructs	CV	H	I	SE	WE
CV					
H	0.827				
I	0.522	0.587			
SE	0.720	0.733	0.554		
WE	0.841	0.817	0.704	0.813	

***Note:*** Concern for Value = CV, Honesty = H, Integrity = I, Sets Example = SE, Work Environment = WE.

In the second stage, honesty, integrity, sets example and concern for values determined subjective norm higher order construct formatively. The higher order construct (i.e. ethical leadership) is formative due to the nature of dimensions, which each represent a different component of the construct and cannot be substituted. Variance Inflation Factor (VIF) used to assess multicollinearity in the indicators of higher order construct. The results (Table-III) exhibit acceptable VIF and significant outer weights for the items of formative construct.

**Table-III:** Results of assessment of measurement model of higher order construct

Construct/Associated Items	Outer Weight	P-value	Multi Collinearity VIF
Ethical Leadership (Formative)			
H	0.192	0.001	2.474
I	0.267	0.000	1.431
CV	0.404	0.000	2.274
SE	0.345	0.000	1.992

***Note:*** Honesty = H, Integrity = I, Concern for Value = CV, Sets Example = SE.

### Assessment of structural model:

**Fig.2:**
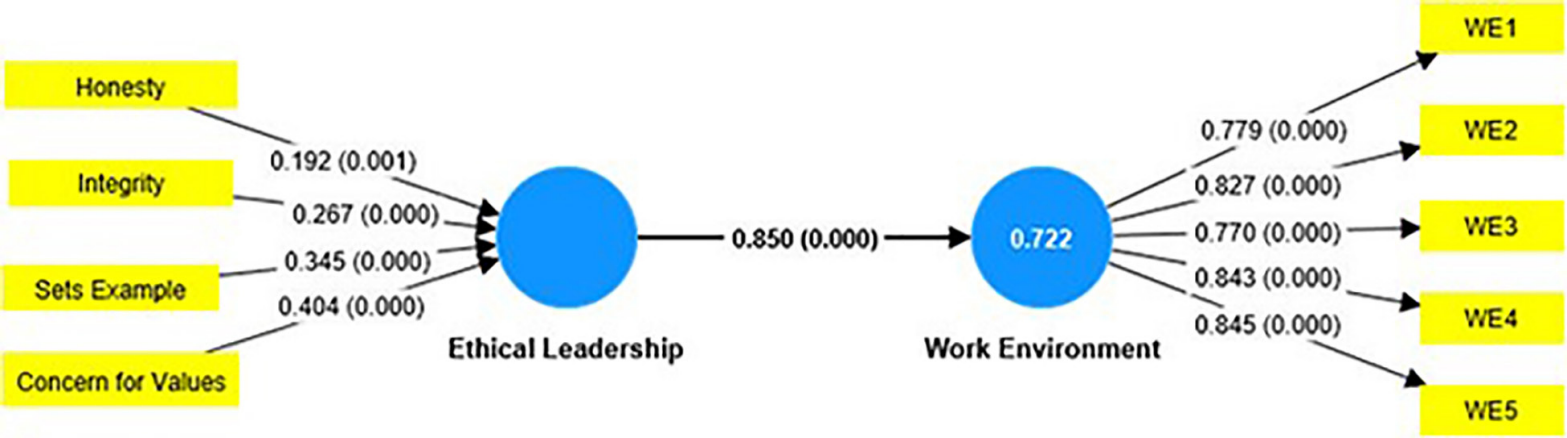
Assessment of Antecedent of Work Environment.

Assessing the proposed hypotheses to validate it is the next step in the structural equation modeling process. Study hypotheses (H_1_) evaluate whether ethical leadership has a significant impact on the work environment. Study results (Table-IV, Fig.2) found statistically significant direct effect of ethical leadership on work environment (β=0.850, t=39.168, P<0.001). Hence, hypothesis (H_1_) was supported.

**Table-IV:** Results of Hypothesis testing.

Hypotheses	Relationship	β	t	P	Decision
H1	EL→WE	0.850	39.168	< 0.000	Supported

***Note 1:*** Ethical Leadership = EL, Work Environment = WE, β = Beta Coefficient, t = t-statistics, P = probability (P) value, *P < 0.001.

### Model’s Explanatory Power:

In order to ascertain models’ explanatory power, R^2^ and predictive relevance (Q^2^) were implied in the current study. The study results found an R^2^ value of 0.722 for the work environment. This shows that a 72.2% variance in the work environment can be attributed to ethical leadership. Based on the recommended value, our model achieved acceptable R^2^ for the work environment is significant. Effect size Q^2^ for predictive relevance of the work environment was 0.850. This showed that the independent variables (i.e. ethical leadership) have a high effect in producing the Q^2^ in the PLS-path model.

## DISCUSSION

The key objective of this study was to assess the impact of ethical leadership on work environment in public hospitals in the context of Saudi Arabia. Our empirical results demonstrate that ethical leadership behavior significantly explains the work environment and contributed to make positive work environment in public hospitals in Hail, Saudi Arabia. Other studies also illustrated and supported the results of this study. It was observed that honesty, integrity, treating subordinates fairly, and honoring moral standards were considered essential attribute of ethical leadership behavior.^24^ A few studies that focused specifically on the group of nurses found a positive relation between ethical leadership and workplace environment.^25^ According to a study conducted in a private German healthcare organization, ethical leadership significantly influences employee satisfaction at workplace.^26^ In addition, Healthcare occupations are well known for being demanding and stressful.^27^ A study undertaken among doctors indicated that stress was positively impacted by ethical leadership.^28^ Ethical leader interacts openly with employees and expresses expectations and duties that should lessen workers’ uncertainty or stress when carrying out their assigned task.^29^ Additionally, an ethical leader who demonstrates honesty, reliability, compassion, and concern for others positively influences workplace environment.^30^

### Limitations of the study:

The sample for the study was not demographically varied. However, it included a variety of public hospitals and professional groups in public hospitals. Hence, the study should be repeated in different healthcare organizations to ensure the representativeness and generalizability of the results. We only looked at a subset of ethical behaviors; thus, it will be crucial for future research to look at how unethical leadership affects a range of ethical behaviors before making clear, practical management recommendations.

## CONCLUSION

In the healthcare sector, the ethical leader creates and supports an environment that encourages high-caliber individuals to act ethically. The results of this study, supported by other studies, indicated that factors like honesty, integrity, sets example, and concern for values are primary characteristics of ethical leadership. The study also revealed that ethical leadership behavior had a positive impact on work environment in public hospital in Hail, Saudi Arabia. Hence, ethical leadership might help an organization to decrease employee stress and fatigue level; decrease the level of relationship conflict among employees; and increase employee satisfaction towards salary and other benefits in the workplace.
